# Experimental Evaluation of the Treatment Effect of High Viscosity Drilling Fluid and Floating Oil Using Ozone Fine Bubble Technology

**DOI:** 10.3390/nano15171324

**Published:** 2025-08-28

**Authors:** Xiaoxuan Guo, Lei Liu, Nannan Liu, Fulong Hu, Lijuan Zhang

**Affiliations:** 1 CNOOC Key Laboratory of Offshore Drilling Fluids and Cementing, Tianjin 300459, China; guoxx6@cosl.com.cn (X.G.); liulei58@cosl.com.cn (L.L.); 2 China Oilfield Services Limited, Tianjin 300459, China; 3 School of Petroleum and Natural Gas Engineering, Changzhou University, Changzhou 213164, China; s24040820025@smail.cczu.edu.cn; 4 Shanghai Institute of Applied Physics, Chinese Academy of Sciences, Shanghai 201800, China; zhanglijuan@sari.ac.cn

**Keywords:** ozone, fine bubbles, drilling fluid, air flotation, high molecular polymer

## Abstract

Drilling fluid plays a critical role in drilling engineering. With the deepening implementation of clean production concepts and increasingly stringent environmental regulations, the treatment standards for drilling wastewater at operational sites have been significantly elevated. In response to the characteristics of high oil content, high COD, high chromaticity, high ammonia nitrogen, and total phosphorus content in drilling, the use of fine bubbles to improve gas utilization efficiency and mass transfer effect, combined with ozone gas, is aimed at degrading difficult-to-degrade high-molecular-weight organic compounds, aiming to solve the problems of high viscosity and high oil content in drilling fluids returned from offshore platforms. Indoor simulation experiments have shown that by using ozone fine bubble technology to treat drilling fluids, the viscosity reduction rate can reach over 29%, and the oil removal rate can reach 40%. Ozone fine bubble technology has significant viscosity reduction and oil removal effects on high viscosity drilling fluids.

## 1. Introduction

Despite the considerable advancements in renewable and alternative energy sources over recent decades, hydrocarbon-based fuels, such as oil and natural gas, are projected to continue serving as the dominant global energy sources for the foreseeable future. Within petroleum engineering, drilling fluids—commonly known as drilling mud—constitute a critical component of oil and gas exploration operations. These fluids fulfill several essential functions, including cooling and lubricating the drill bit, transporting and suspending drill cuttings, stabilizing the wellbore to prevent collapse, transmitting hydraulic energy to downhole tools, and protecting hydrocarbon reservoirs from damage [1,2,3,4,5]. Various types of drilling fluids are employed to facilitate efficient drilling processes. Conventional additives, such as xanthan gum, polyanionic cellulose, and bentonite, are widely used to enhance rheological properties and mitigate fluid loss. Nevertheless, their performance remains limited under extreme operational conditions [6,7,8,9]. Moreover, these additives contribute substantially to drilling waste volumes. The treatment and reclamation of drilling fluids have thus emerged as significant research topics, bearing considerable importance for environmental protection and sustainable resource utilization [10]. Drilling fluids are complex mixtures comprising high-molecular-weight polymers (e.g., xanthan gum and starch), nano-sized solid particles, salts, alkalis, and hydrocarbon phases. They are typically characterized by high oil content, elevated chemical oxygen demand (COD), intense chromaticity, and significant concentrations of ammonia nitrogen and total phosphorus [11]. Conventional treatment methodologies often involve oxidative degradation of polymers using strong oxidants, followed by solid–liquid separation through centrifugation, flocculation, and filtration. The treated liquid phase may then be recycled [12,13]. However, these approaches are associated with several limitations, including handling risks of strong oxidants, spatial constraints on offshore platforms, and prolonged separation cycles [14]. Such challenges highlight persistent technical bottlenecks in the treatment of oily drilling fluids, both onshore and offshore. There is a compelling need for innovative technologies capable of efficiently degrading refractory polymers, mitigating residual oil droplet pollution, and improving processing efficiency [14,15].

Research on drilling fluids constitutes a highly multidisciplinary domain that integrates principles and methodologies from materials science, petroleum engineering, chemistry, and environmental science to tackle the complex challenges inherent in modern drilling operations [16]. The primary strategies for managing oily drilling fluids include reinjection into subsurface formations, recycling for reuse, and treatment followed by discharge in compliance with environmental regulations [17]. The fundamental principle of reinjection involves preliminary treatment of drilling fluids—through processes such as oil removal, coagulation-sedimentation, air flotation, and filtration—prior to their injection into deeper geological formations. This approach significantly mitigates environmental pollution [18]. Key considerations during reinjection include compatibility with formation water, concentration of suspended solids, and residual oil content, with specific water quality requirements being highly dependent on local geological conditions and operational parameters. The recycling strategy focuses on the recovery of valuable components from drilling fluids, with the treated aqueous phase being reused in the formulation of new drilling fluids. For instance, Qiu Chunsheng et al. employed an oxidation–ion capture flocculation process to treat drilling wastewater, demonstrating that the reclaimed water had negligible adverse effects on the performance of recycled drilling fluids [19]. Alternatively, the discharge approach necessitates treating waste drilling fluids to comply with stringent environmental regulations, such as the first-level criteria stipulated in the “Integrated Wastewater Discharge Standard” (GB 8978-1996) [20], prior to release into the environment.

Due to stringent environmental regulations and operational constraints, offshore platforms are compelled to prioritize resource-oriented management of returned drilling fluids. This entails the recovery of residual petroleum from wastewater and the recycling of treated water for reuse. Fine bubbles, defined as gaseous dispersions at the micrometer and nanometer scale, exhibit distinctive physicochemical characteristics—including a high specific surface area, extended residence time in aqueous media, and significant surface charge properties—which enhance their effectiveness in advanced treatment processes. Due to their unique physicochemical properties, fine bubbles are currently mainly applied in drinking water, medicine, and plant cultivation. The research and application of fine bubble technology in the fields of cultivation and photovoltaic cell manufacturing can help improve production efficiency, enhance product quality, and promote sustainable development in related industries. It has good promotion and application market potential. However, the application of fine bubble technology in the field of oil fields is relatively limited. Wang R Q et al. [21] conducted experiments on treating oil and gas field drilling wastewater using ozone fine bubble technology. During ozonation, ozone (O_3_) can exhibit synergistic effects when combined with specific substances or reagents, leading to the generation of active oxygen species that effectively decolorize returned drilling fluids. Furthermore, advanced oxidation processes (AOPs) facilitate the removal of certain soluble organic contaminants, underscoring the broad applicability of ozone-based technologies in the treatment of oil and gas field wastewater. Building upon the mechanisms of ozone oxidation and the unique attributes of fine bubbles, this study integrates ozone with fine bubble technology to enhance the elimination of soluble organic matter from aqueous systems. The introduction of ozone in the form of fine bubbles significantly improves mass transfer efficiency. Moreover, the interfaces and collapse of these fine bubbles promote the formation of hydroxyl radicals (·OH) [22], which catalyze chain reactions between ozone and organic compounds. This process not only accelerates the oxidation rate but also operates without generating exhaust gases, offering both economic and environmental benefits. By incorporating fine bubble technology into chemically advanced oxidation, this approach enables more efficient and cost-effective degradation of high-molecular-weight polymers present in high-viscosity drilling fluids while simultaneously minimizing secondary pollution. Hence, there is a compelling need to investigate the application of ozone fine bubble technology for treating high-viscosity drilling fluids, to quantitatively assess its efficacy in viscosity reduction and oil removal, and to establish a novel, efficient, economical, and sustainable treatment methodology. Such advancements are critical to driving the evolution and implementation of improved treatment technologies for recovered drilling fluids.

## 2. Materials and Experiments

### 2.1. Experimental Materials

The primary chemicals utilized in the preparation of the drilling fluid were sourced directly from operational drilling sites. The fundamental constituents of the fluid formulation included PF-EZVIS, PF-EZFLO, PF-EZCARB, PF-JLX C, and PF-LUBE. Two distinct types of drilling fluids were formulated: a base drilling fluid (denoted as 1#) and an oil-contaminated variant (denoted as 2#), prepared by introducing crude oil into the base formula. Additional reagents—namely sodium hydroxide, sodium chloride, magnesium chloride, calcium chloride, potassium chloride, sodium carbonate, sodium bicarbonate, polyaluminum chloride, polyacrylamide, and sodium sulfate—were employed, all of which were of analytical grade.

Among these components, PF-EZVIS is a high-molecular-weight polysaccharide polymer presented as a light yellow free-flowing powder; PF-EZFLO functions as a fluid loss reducer and exhibits a white free-flowing powder form; PF-EZCARB consists of a graded mixture of calcium carbonate particles and appears as a white free-flowing powder; both PF-JLX C and PF-LUBE serve as lubricants and are supplied as light yellow viscous liquids.

Following the preparation of the drilling fluid in accordance with the specified formulation, key parameters of the fluid were measured. The corresponding results are summarized in Table 1.

### 2.2. Experimental Equipment

**Figure 1:**
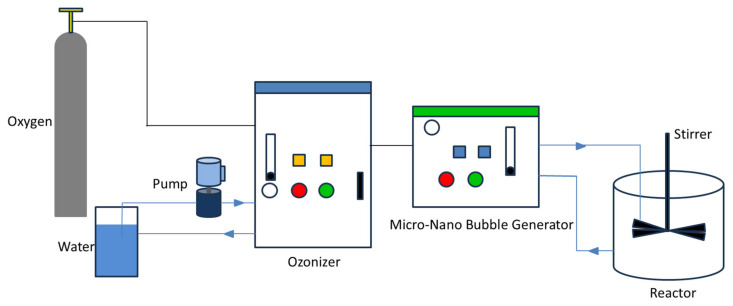
Equipment.

The experimental setup utilized in this study is schematically illustrated in Figure 1. The fine bubble generator was custom-designed and fabricated in the laboratory, operating on a principle that integrates a Venturi tube with an aeration disk. The ozone generator was used to produce high-concentration ozone gas from input oxygen. The fine generator was then used to dissolve and disperse this ozone gas into the aqueous phase, creating a mixture of ozone fine bubbles in water. Other relevant instruments used in the experiment are listed in Table 2.

### 2.3. Experimental Methods

This study is designed to investigate the efficacy of ozone fine bubble technology in removing organic pollutants from high-viscosity drilling fluids, assess its potential for practical application in the treatment of such fluids, and ultimately address the challenges posed by elevated viscosity and high oil content. The experimental work was structured into two distinct phases:

(1) To validate the effectiveness of ozone fine bubble technology for treating high-viscosity drilling fluids, laboratory experiments were conducted on fluid samples. The evaluation focused on quantifying the viscosity reduction efficiency and assessing the feasibility for field applications.
(2) To enhance residual oil removal, a combined process was implemented involving flocculation pretreatment followed by ozone fine bubble flotation. The efficacy of this treatment was determined through quantitative analysis of the residual oil content in the processed drilling fluid samples.

#### 2.3.1. Viscosity Reduction Treatment of Original Drilling Fluid Using Ozone Fine Bubbles

The experimental procedure for viscosity reduction treatment of the base drilling fluid (1#) using ozone fine bubbles was conducted as follows: first, the gas line of the ozone generator and the circulating water-cooling circuit were connected and purged to eliminate residual gases within the system. Then, 500 mL of the base drilling fluid was introduced into the reaction vessel under mechanical stirring. The current of the ozone generator was adjusted to achieve the target ozone concentration. Upon completion of the predetermined reaction time, the power and gas supply were terminated, and tail gas was properly managed throughout the experiment. Samples were collected using a syringe from a designated sampling point and transferred to reagent bottles for subsequent analysis.

#### 2.3.2. Evaluation of Oil Removal from Oil-Containing Drilling Fluid Using Ozone Fine Bubbles

The experimental procedure for oil removal treatment of the oil-containing drilling fluid (2#) using ozone fine bubbles is as follows: first, add the pre-weighed flocculant to the reaction container. After the set flocculation time, connect the gas path of the fine bubble generator, adjust the gas flow rate, and turn on the device to start generating fine bubbles. When the set reaction time is reached, disconnect the power and close the gas path. Remove the floating scum from the upper layer, and the remaining liquid is the treated oil-containing drilling fluid. Use a syringe to sample at a fixed position and transfer the sample to a reagent bottle for subsequent testing.

### 2.4. Detection Methods

pH was measured according to GB/T 6920-1986 [23] “Water Quality—Determination of pH—Glass Electrode Method.” Viscosity was determined in accordance with GB/T 29170-2012 [24] “Petroleum and natural gas industries—Laboratory testing of drilling fluids”. Oil content was determined using reagents and methods referenced from the standard “Water Quality—Determination of Petroleum and Animal and Vegetable Oils—Infrared Spectrophotometry.” Gaseous ozone concentration was measured according to GB/T 37894-2019 [25] “Technical Requirements for Ozone Generators in Water Treatment—Ultraviolet Absorption Method.”

## 3. Results and Discussion

### 3.1. Evaluation of Viscosity Reduction Effect Using Ozone Fine Bubbles

**Figure 2:**
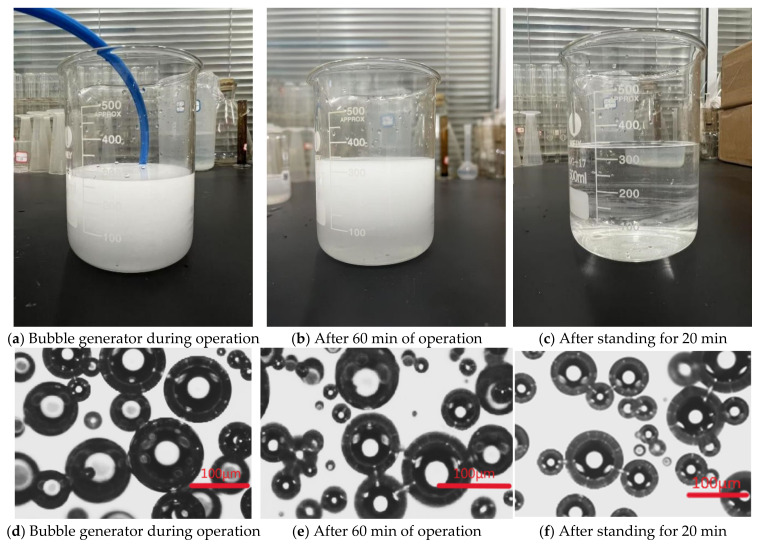
Experimental site images and images collected by laser confocal microscopy.

Prior to the formal experiments, the stability and generation efficiency of fine bubbles were evaluated. A 300 mL sample of ultrapure water was subjected to fine bubble generation for 60 min at a gas intake flow rate of 90 mL/min. Samples were collected immediately after generation for initial analysis. The same batch was subsequently allowed to stand for 20 min at ambient conditions, followed by immersion in a 50 °C water bath for 20 min, and finally stored at 4 °C for an additional 20 min before repeating sampling and testing. Representative experimental diagrams and processed bubble images, obtained via microscopy and analyzed using ImageJ (1.8.0) software, are presented in Figure 2.

**Figure 3:**
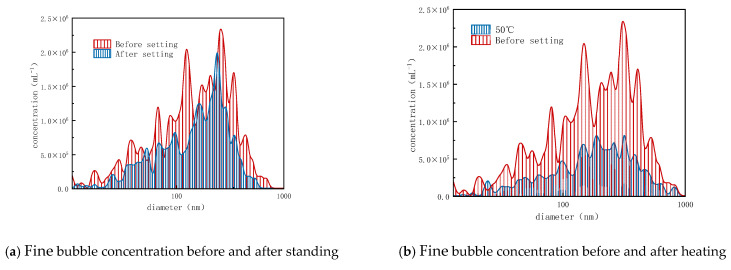

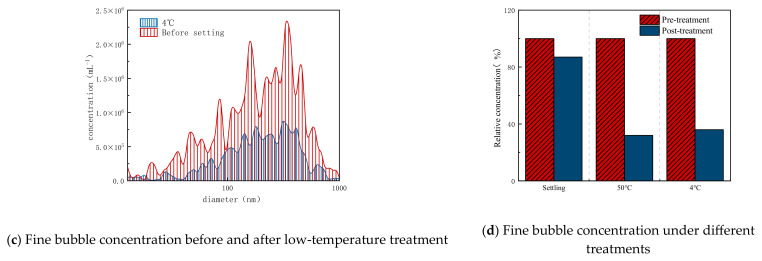
The variation in nanobubble concentration with temperature.

The experimental results are shown in Figure 3. Comparing the changes in fine bubbles after standing for 20 min, heating and low temperatures significantly disrupted the bubbles, indicating that temperature changes severely affect bubble stability, causing fine bubbles to rapidly dissipate.

**Figure 4:**
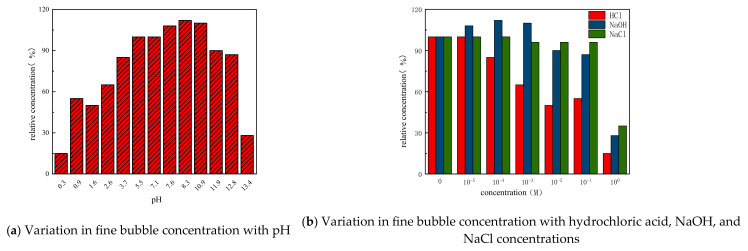
The variation in nanobubble concentration with pH, hydrochloric acid, NaOH, NaCl concentration.

In addition to temperature, the influences of other environmental conditions on fine bubbles were systematically examined. Hydrochloric acid (HCl) and sodium hydroxide (NaOH) were employed to modulate pH levels, while sodium chloride (NaCl) was introduced to evaluate the impact of ionic strength. Samples were collected and analyzed five minutes after the addition of each reagent at varying concentrations. A comprehensive summary of the effects of acid, base, salt concentration, and pH on fine bubble stability is provided in Figure 4. From a pH-dependency standpoint, fine bubble stability was markedly compromised under both highly acidic and strongly alkaline conditions. In contrast, weakly alkaline environments resulted in greater stability compared to neutral conditions. With regard to reagent concentration, elevated levels of acids, bases, and salts uniformly impaired bubble integrity. At lower concentrations, however, only acids exhibited a destabilizing effect, whereas bases and salts demonstrated negligible influence.

**Figure 5:**
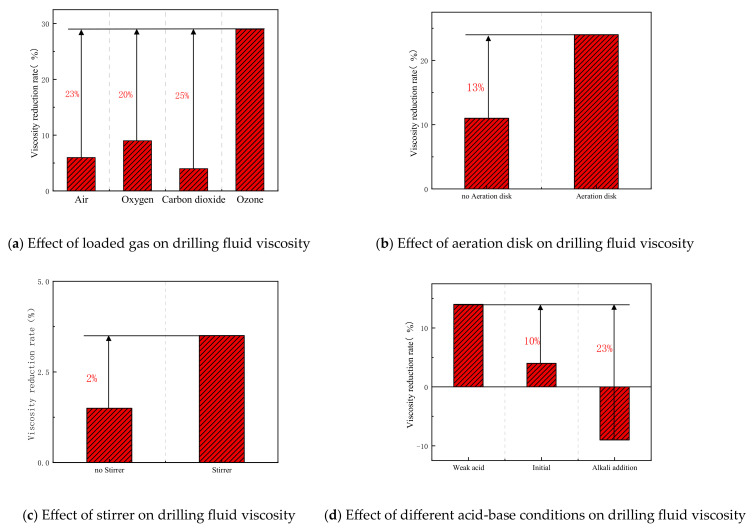
Changes in viscosity of drilling fluid due to loading gas, aeration disk, agitator, and acidity/alkalinity.

The effects of different gases (air, oxygen, carbon dioxide, and ozone) on the degradation of high-molecular-weight polymers were examined. The effects of ozone introduction with and without an aeration disk, and with and without a stirrer, on polymer degradation were studied. The effects of reactions under weakly acidic, original, and alkaline conditions were also investigated. Other conditions were as follows: total reaction time of 4 h, gas introduced at 500 mL/min for 1 h via an aeration disk, temperature of 26 °C, low-speed stirring at 300 rpm during the reaction, followed by high-speed stirring at 10,000 rpm for 20 min, and viscosity testing. The results are shown in Figure 5. Ozone outperformed other gases, achieving a viscosity reduction rate of 29%. This indicates that the type of gas loaded affects the degradation of high-molecular-weight polymers to some extent, as the physical and chemical properties of the gas, such as solubility, diffusivity, compressibility, and molecular weight, influence the performance of fine bubbles. The conclusion is that ozone is more effective than other gases, and both the aeration disk and stirrer aid in viscosity reduction, with better results under acidic conditions than under alkaline conditions.

**Figure 6:**
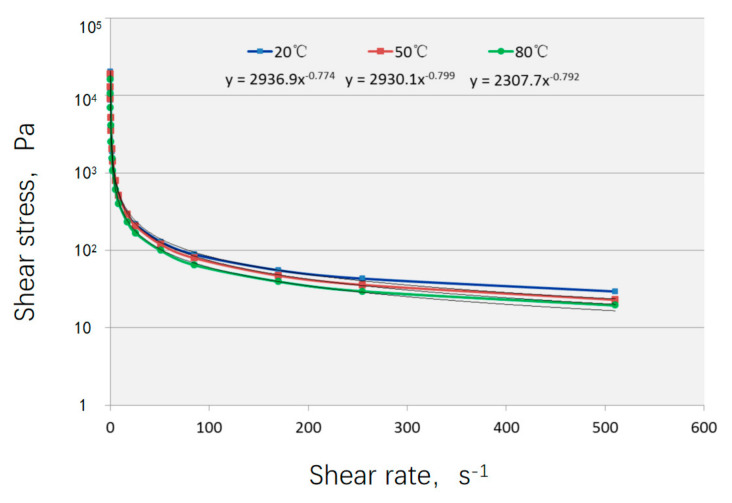
The relationship between shear stress and shear rate of the drilling fluid obtained from field tests.

**Figure 7:**
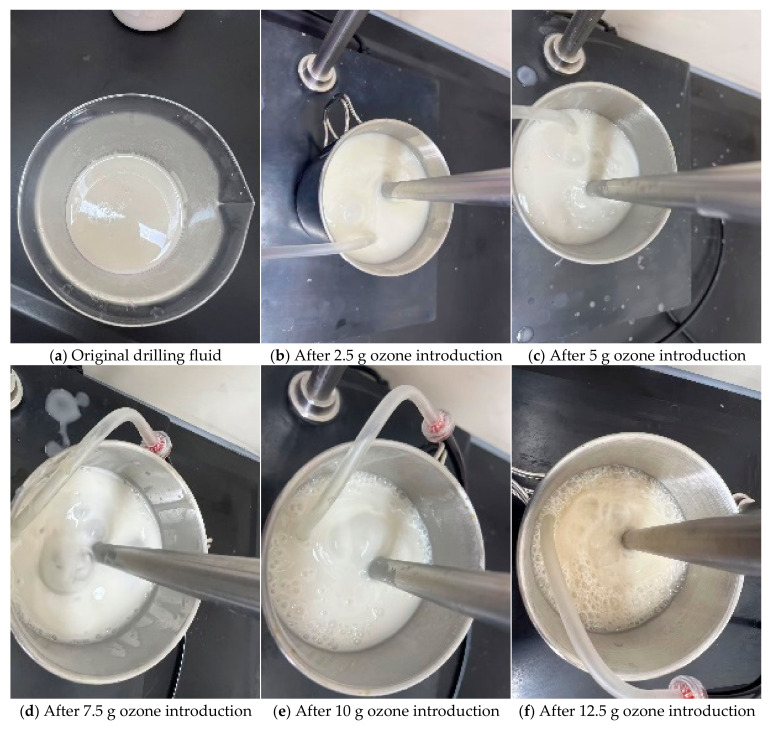
Drilling fluid changes with ozone dosage.

The original drilling fluid remained a milky white viscous liquid before and after ozone introduction. The non-Newtonian viscosity of the drilling fluid measured on site is shown in Figure 6. The shear stress decreases rapidly with the increase in shear rate. As the ozone dosage increased, the viscosity of the drilling fluid continuously decreased. When the ozone dosage reached 7.5 g, bubbles rising from the bottom appeared and burst on the surface, and the drilling fluid gradually became thinner. During the cumulative ozone introduction of 7.5 g, the solution viscosity remained high. At 9 g, the viscosity significantly decreased, and residual bubbles were observed at the interface. From 9 g to 12.5 g, the viscosity reduction was no longer significant, the solution became more fluid, and a large number of residual bubbles were observed in the upper layer of the drilling fluid, as shown in Figure 7.

**Figure 8:**
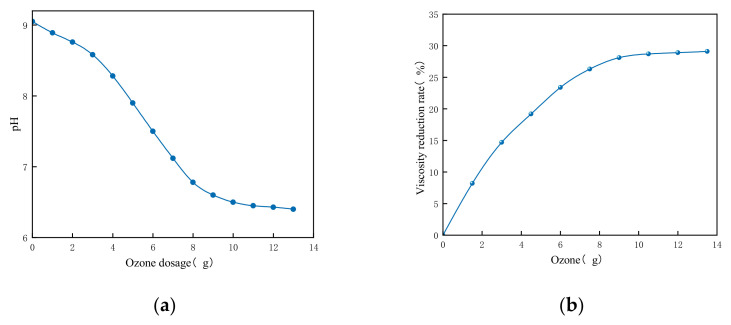
Trend of pH and viscosity changes during the reaction process.

As illustrated in Figure 8a, the initial pH of the drilling fluid measured 9.1. Following the initiation of ozone injection, the pH gradually decreased to 8.3, then declined rapidly to 6.4 with continued ozonation, and stabilized thereafter for the remainder of the experiment. Correspondingly, as indicated in Figure 8b, a viscosity reduction of 29% was achieved at an accumulated ozone dosage of 12.5 g.

As shown in Figure 8a,b, when the ozone dosage was below 10 g, both the pH and the viscosity of the drilling fluid decreased continuously, indicating effective oxidation of organic components by ozone. Once the ozone dosage exceeded 10 g, the rate of viscosity reduction slowed and the pH stabilized, suggesting the conclusion of the organic oxidation phase and the onset of mineralization. These results demonstrate that ozone fine bubbles can directly degrade organic matter in drilling fluids without the need for solid catalysts. This degradation mechanism is attributed to the decomposition of ozone under alkaline conditions, yielding hydroxyl radicals ( OH) that non-selectively oxidize and break down high-molecular-weight polymers at an accelerated reaction rate [26,27,28,29]. Furthermore, previous studies have confirmed that the combination of ozone with fine bubbles enhances the generation of ·OH [30,31], thereby promoting the catalytic degradation of refractory organic substances in drilling fluids.

### 3.2. Evaluation of Oil Removal Effect Using Ozone Fine Bubbles

**Figure 9:**
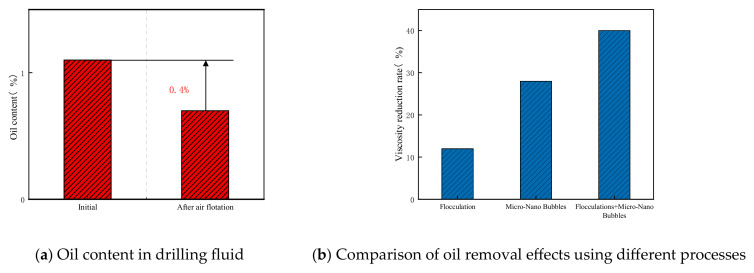
Oil removal test using different processes.

A total of 850 mL of the oil-based drilling fluid (2#) was treated using an aeration disk at 26 °C, with air introduced at a flow rate of 500 mL/min for 2 h. The upper oil layer was skimmed at 10-min intervals throughout the process. Upon completion of aeration, the mixture was stirred at 10,000 rpm for 20 min, and a 10 mL aliquot of the treated fluid was transferred to a separatory funnel. Three consecutive extractions were carried out using n-hexane. The combined extracts were dehydrated by filtration through a Buchner funnel containing anhydrous sodium sulfate, and the solvent was subsequently evaporated at 60 °C using a rotary evaporator. The experimental results are summarized in Figure 9a. The initial oil content of the drilling fluid was 1.1%, based on theoretical calculation. After air flotation treatment, the measured oil content was reduced to 0.7%. As further illustrated in Figure 9b, the integration of flocculation with fine bubble technology achieved an overall oil removal efficiency of 40%.

**Figure 10:**
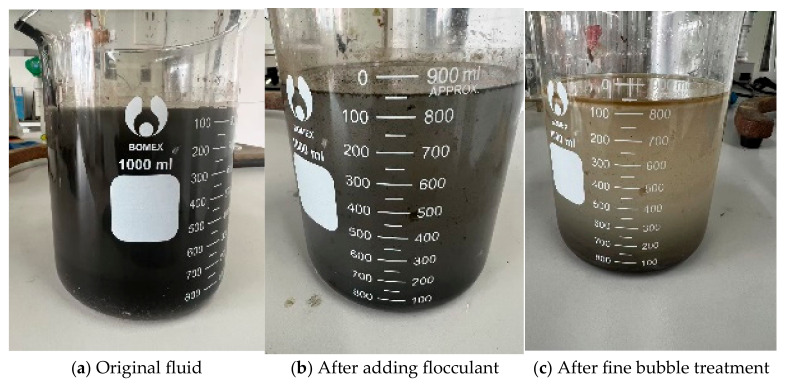
Water quality changes with ozone dosage.

Based on experimental observations, the introduction of organic high-molecular-weight flocculants induced particle aggregation and growth in the drilling fluid through adsorption and bridging mechanisms. Concurrently, controlled mechanical stirring enhanced phase separation efficiency [32,33]. Upon flocculant addition, solid particles were rapidly driven toward the container bottom by centrifugal force generated during rotation, forming a distinct solid sediment layer and enabling efficient mud–water separation. As depicted in Figure 10b, the flocculated drilling fluid exhibited clear stratification. Figure 10c further reveals a well-defined three-layer structure: floating oil in the upper phase, water in the intermediate layer, and concentrated solid precipitates at the bottom. It is critical to optimize operational parameters during drilling fluid treatment. Excessive dosages of PAC or PAM, prolonged flocculation stirring time, or elevated stirring speeds may impair separation performance [34]. Beyond threshold values, these parameters adversely affect flocculation efficacy [35]. Intense or prolonged stirring can fragment large flocs into finer particles, elevating suspended solid content in the effluent. Overdosing of flocculants may also increase aqueous phase viscosity and turbidity. Therefore, precise control of experimental conditions is essential to achieve optimal treatment outcomes.

## 4. Conclusions

(1) Ozone fine bubble technology demonstrates significant efficacy in reducing viscosity and removing oil content from high-viscosity drilling fluids.
(2) Deep treatment using this technology achieved a 29% reduction in viscosity at an ozone dosage of 12.5 g, while the combination of flocculation and fine bubble treatment resulted in a 40% oil removal rate.

Given that drilling fluids contain substantial refractory organic constituents, ozone fine bubble technology offers a catalyst-free method for degrading these recalcitrant compounds, thereby facilitating advanced treatment of waste fluids at drilling sites.

## Figures and Tables

**Table 1:** Reagent Parameters.

Formula No.	pH	AV (mPa·s)	PV (mPa·s)	YP (Pa)
1#	8–10	24–27	8–22	10–24
2#	8–10	26–30	10–21	10–25

**Table 2:** Experimental instruments and models.

Instrument Name	Model	Manufacturer
Ultrapure Water System	YK-RO-B	Xiamen Shuhuoquan
Ozone Generator	ZJC-TF10	Shanghai Zhongjing
Hot Rolling Oven	XGRL-5	Qingdao Xinruide
High-Speed Stirrer	GJS-B12K	Qingdao Xinruide
Electronic Balance	WTB10002K	Changzhou Wantai
Six-Speed Viscometer	ZNN-D6	Qingdao Xinruide
Electric Stirrer	D90-150	Qingdao Xinruide
Magnetic Stirring Water Bath	HH-2J	Changzhou Langyue
Digital Constant Temperature Bath	HH-6	Shanghai Lichen Bangxi
